# Tumour vascularity is a significant prognostic factor for cervix carcinoma treated with radiotherapy: independence from tumour radiosensitivity

**DOI:** 10.1038/sj.bjc.6690700

**Published:** 1999-09

**Authors:** R A Cooper, C M L West, D P Wilks, J P Logue, S E Davidson, S A Roberts, R D Hunter

**Affiliations:** CRC Section of Genome Damage and Repair Biomathematics and Computing Unit, Paterson Institute for Cancer Research Department of Clinical Oncology, Christie Hospital NHS Trust, Wilmslow Road, Manchester, M20 4BX, UK

**Keywords:** angiogenesis, SF2, predictive assays

## Abstract

The aim of the study was to investigate the relationship between intrinsic radiosensitivity and vascularity in carcinoma of the cervix given radiotherapy, and assess whether more refined prognostic information can be gained by combining the two parameters. A retrospective study was carried out on 74 patients with locally advanced carcinoma of the cervix. Formalin-fixed, paraffin-embedded tumour biopsies were stained with anti-factor VIII using immunohistochemistry. Vascularity was scored using the intra-tumour microvessel density (IMD), or ‘hot-spot’, technique. For the same patients, the measurement of intrinsic radiosensitivity (SF2) had been made previously on the same pretherapy samples. Patients were stratified by the median IMD and SF2 scores. Women with radioresistant and highly vascular tumours had poorer 5-year survival (*P* = 0.0005, *P* = 0.035 respectively) and local control (*P* = 0.012, *P* = 0.077 respectively) than those with radiosensitive and poorly vascular tumours. No significant correlation was seen between IMD and SF2. Multivariate analysis (including tumour stage and patient age) showed that only SF2 and IMD were significant prognostic factors for survival. Patients with both a radioresistant and highly vascular tumour had a 5-year survival level of 18% compared to 77% for those patients with a radiosensitive and poorly vascularized tumour. Tumour angiogenesis and cellular radiosensitivity are independent prognostic factors for cervix carcinoma treated with radiotherapy. Allowing for tumour radiosensitivity increases the prognostic significance of vascularity measurements in cervix tumours. © 1999 Cancer Research Campaign


*British Journal of Cancer* (1999) **81**(2), 354–358
© 1999 Cancer Research Campaign
Article no. bjoc.1999.0700

### Tumour vascularity is a significant prognostic factor for cervix carcinoma treated with radiotherapy: independence from tumour radiosensitivity

**RA Cooper[1], CML West[1], DP Wilks[1], JP Logue[3], SE Davidson[3], SA Roberts[2] and RD Hunter[3]**

[1]CRC Section of Genome Damage and Repair and [2]Biomathematics and Computing Unit, Paterson Institute for Cancer Research, [3]Department of Clinical Oncology, Christie Hospital NHS Trust, Wilmslow Road, Manchester M20 4BX, UK

**Summary** The aim of the study was to investigate the relationship between intrinsic radiosensitivity and vascularity in carcinoma of the cervix given radiotherapy, and assess whether more refined prognostic information can be gained by combining the two parameters. A retrospective study was carried out on 74 patients with locally advanced carcinoma of the cervix. Formalin-fixed, paraffin-embedded tumour biopsies were stained with anti-factor VIII using immunohistochemistry. Vascularity was scored using the intra-tumour microvessel density (IMD), or 'hot-spot', technique. For the same patients, the measurement of intrinsic radiosensitivity (SF2) had been made previously on the same pretherapy samples. Patients were stratified by the median IMD and SF2 scores. Women with radioresistant and highly vascular tumours had poorer 5-year survival ($P = 0.0005$, $P = 0.035$ respectively) and local control ($P = 0.012$, $P = 0.077$ respectively) than those with radiosensitive and poorly vascular tumours. No significant correlation was seen between IMD and SF2. Multivariate analysis (including tumour stage and patient age) showed that only SF2 and IMD were significant prognostic factors for survival. Patients with both a radioresistant and highly vascular tumour had a 5-year survival level of 18% compared to 77% for those patients with a radiosensitive and poorly vascularized tumour. Tumour angiogenesis and cellular radiosensitivity are independent prognostic factors for cervix carcinoma treated with radiotherapy. Allowing for tumour radiosensitivity increases the prognostic significance of vascularity measurements in cervix tumours.

© 1999 Cancer Research Campaign

**Keywords:** angiogenesis; SF2; predictive assays

Radiotherapy is the main treatment modality for locally advanced carcinoma of the cervix (Eifel et al, 1993). However, outcome, stage for stage, has not changed over the past 25 years and is particularly poor for later stage disease (Lindegaard et al, 1996). It has been suggested that a better understanding of the biology of cervix tumours may lead to the definition of more refined prognostic groups, which in turn may lead to new therapeutic interventions (Kapp et al, 1996). Parameters such as stage and tumour volume are currently the most established indicators of radiotherapy response. However, within these prognostic divisions there is a large variation in response to treatment.

Recently, there has been increased interest in the assessment of tumour angiogenesis as a potential prognostic factor. This stems from studies that have established the essential role of angiogenesis in tumour growth and progression (Folkman, 1990). Angiogenesis is generally assessed as tumour vascularity, and in carcinoma of the cervix a number of different measurement techniques have been used. Older studies used inter-capillary distance (Kolstad, 1968; Awwad et al, 1986) or percentage of endothelial cells (Siracka et al, 1982) and found that low-vascular density was significantly associated with poor outcome. However, a recent consensus statement proposed the intra-tumour microvessel density (IMD) ('hot-spot') technique as the method of choice for measuring angiogenesis (Vermeulen et al, 1996). Using this technique, in the majority of studies, high vascularity has been found to correlate with poor outcome in a number of different tumour sites including carcinoma of the cervix (Wiggens et al, 1995; Dinh et al, 1996; Weidner et al, 1996). Using the IMD method, we have shown recently that patients with well vascularized tumours have a significantly poorer survival ($P = 0.038$) and local control ($P = 0.028$) than patients with poorly vascularized tumours (Cooper et al, 1998).

In carcinoma of the cervix, the surviving fraction after 2 Gy (SF2) has been found not only to be an important predictor of outcome following radiotherapy, but also to be independent of other prognostic factors (West et al, 1993, 1997). Based on mathematical modelling it has been proposed that measurements of tumour radiosensitivity are most likely to correlate with clinical outcome following radiotherapy (Tucker et al, 1989). It was suggested, in that paper, that it may be necessary to correct for intrinsic radiosensitivity before predictive assays based on other factors might have any clinical significance. The aim of this paper therefore was to explore the relationship between intrinsic radiosensitivity and vascularity and to establish whether allowing for radiosensitivity increased the prognostic significance of vascularity measurements in carcinoma of the cervix.

*Received 3 December 1998*
*Revised 18 March 1999*
*Accepted 22 March 1999*

*Correspondence to:* CML West

### METHODS AND MATERIALS

### Patients

Table 1 summarizes the patient characteristics. Seventy-four patients with cervical carcinoma undergoing radical radiotherapy were included in the study. The patients comprised a consecutive

**Table 1** Patient characteristics

| | Characteristics | Number |
|---|---|---|
| Stage[a] | I | 24 |
| | II | 31 |
| | III | 19 |
| Histology | SCC[b] well[c] | 13 |
| | mod[c] | 41 |
| | poor[c] | 17 |
| | Adeno[d] | 3 |
| Maximum diameter | < 4 cm | 20 |
| | ≥ 4 cm | 22 |

[a]According to the FIGO staging system. [b]Squamous cell carcinoma. [c]Well, moderately and poorly differentiated. [d]Adenocarcinoma.

series for whom both vascularity and radiosensitivity measurements were available. Patient ages ranged from 23 to 85 years with a mean of 53 years. The majority of tumours were squamous cell carcinoma and three were adenocarcinoma. All patients were included in analyses and the results of the study were unaltered when the three adenocarcinomas were excluded. Information on the maximum tumour diameter was only available for 42 of the patients.

Radical radiotherapy was given according to the doses and schedules of the Manchester School of Radiotherapy (Hunter, 1991). All patients gave prior informed consent. Follow-up of patients was 3-monthly in the first 2 years, 4-monthly in the third year and 6-monthly until 5 years. The median follow-up time in surviving patients was 86 months, range 28–106. Suspected tumour recurrence was confirmed either radiologically, histologically or both.

### Measurement of tumour vascularity

Formalin-fixed, paraffin-embedded histological sections, 5-μm thick, were cut from the biopsy specimens. Samples were stained for anti-factor VIII (Dako) as previously described (Davidson et al, 1994). Vascularity was assessed using the 'hot-spot' technique (Weidner et al, 1991). First, the whole tumour section was scanned at low power (× 120) to identify the region of most intense neovascularization. Within this region the number of vessels in three separate random fields were counted at high power (× 25 objective and × 12 ocular; field size 0.142 $mm^2$). The mean number of vessels from the three fields was used in the analysis. Any brown-staining endothelial cell or group of cells, clearly separate from adjacent tumour or stromal cells, was considered a single countable vessel. Areas of gross haemorrhage or necrosis were avoided. Scoring was performed by a single observer without prior knowledge of patient outcome. Intra-observer reproducibility was assessed by scoring ten randomly chosen sections twice. Inter-observer variability was assessed by scoring 20 randomly chosen sections by two independent scorers.

### Measurement of tumour radiosensitivity

Intrinsic radiosensitivity was determined as sensitivity to a single in vitro 2 Gy dose of radiation (SF2). Tumour was disaggregated using an enzyme cocktail prior to irradiation with a $^{137}Cs$ γ-source at a dose rate of 3.8–4.2 Gy $min^{-1}$. Single-cell suspensions were

**Table 2** Cox multivariate analysis showing the relative risk (RR) for 74 cervix tumours

| | Survival | | Local Control | |
|---|---|---|---|---|
| Variable | RR (95% CI) | *P* | RR (95% CI) | *P* |
| SF2 | 3.9 (1.8–8.4) | 0.001 | 3.7 (1.4–9.8) | 0.008 |
| IMD | 2.7 (1.3–5.6) | 0.007 | 3.2 (1.2–8.4) | 0.017 |
| Age | NS | 0.079 | NS | 0.96 |
| Stage | NS | 0.90 | NS | 0.57 |

NS = not significant.

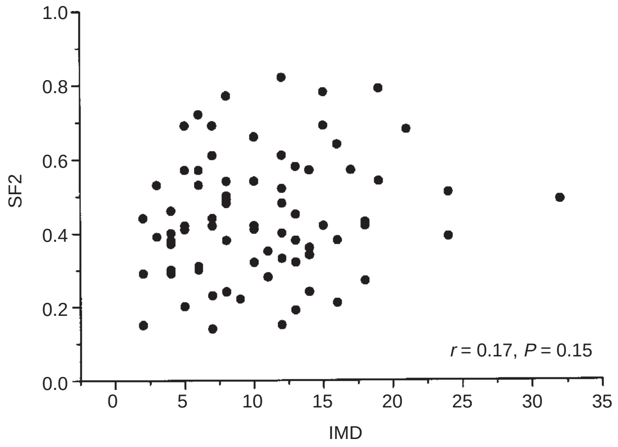

**Figure 1** The relationship between SF2 and IMD for 74 patients with carcinoma of the cervix cultured using a soft agar clonogenic assay and the SF2 calculated from the colony forming efficiencies of control or irradiated samples after 4 weeks growth (West et al, 1993). Characterization of cells growing in colonies was carried out using immunohistochemistry with CAM5.2 and CK1 antibodies to confirm that the colonies grown were of malignant epithelial cell origin (West et al, 1993).

### Statistics

The relationship between variables was investigated using Spearman's correlation coefficient. Overall survival and local control probabilities were determined using univariate and bivariate (stratified) log-rank analysis, stratifying by the parameter medians. Values falling on medians meant that the numbers in each group were unequal. A step-wise Cox multivariate regression analysis was also performed. A significance level of $P = 0.05$ was used throughout.

### RESULTS

### Vascularity and outcome

Using the IMD technique we have previously shown a high level of correlation between repeat measurements by the same ($r = 0.84$, $P = 0.04$), and two independent ($r = 0.88$, $P < 0.0001$) scorers (Cooper et al, 1998). The median IMD for the 74 patients was 10 (range 2–32). Patients were stratified into two groups, those with a

© 1999 Cancer Research Campaign

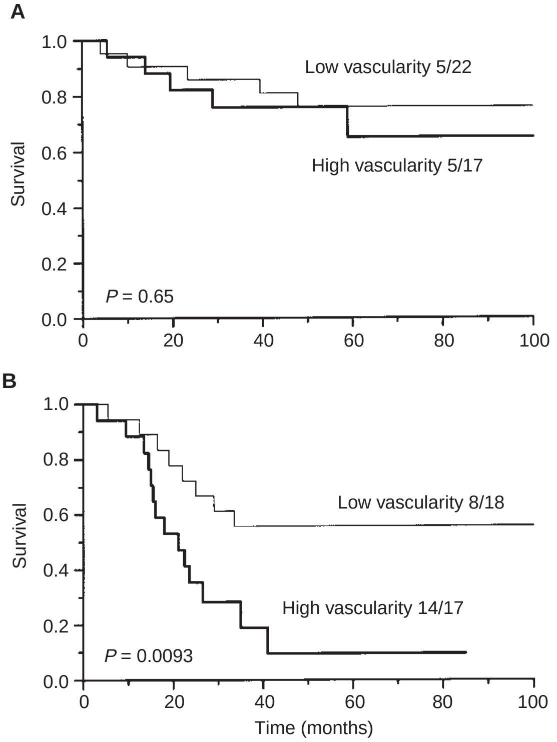

**Figure 2** Survival in respect to vascularity for: (**A**) patients with radiosensitive tumours and (**B**) patients with radioresistant tumours. The values above each arm of the graph indicate the number of deaths/patients in each group. Patients were stratified by the overall median values for both SF2 (of 0.42) and vascularity (of 10)

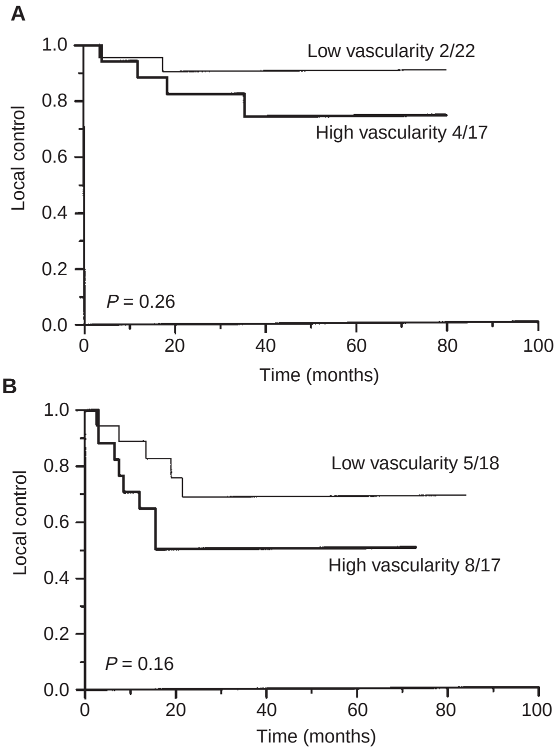

**Figure 3** Local control in respect to vascularity for: (**A**) patients with radiosensitive tumours and (**B**) patients with radioresistant tumours. The values above each arm of the graph indicate the number of local recurrences/patients in each group. Patients were stratified by the overall median values for both SF2 (of 0.42) and vascularity (of 10)

mean IMD > 10 (high vascularity tumours) and those with a mean IMD ≤ 10 (low vascularity tumours). The 5-year survival rate for patients with low vascularity tumours was 77% compared to 44% for those with high vascularity tumours ($P = 0.035$). The 5-year local control rate was 83% and 65% for patients with low and high vascularity tumours respectively ($P = 0.077$).

### SF2 and outcome

The median SF2 value for the 74 patients was 0.42 (range 0.14–0.82). Patients were divided into those with radiosensitive tumours, SF2 ≤ 0.42 and those with radioresistant tumours SF2 > 0.42. Patients with radiosensitive tumours had a significantly higher 5-year survival rate of 74% compared to 37% for those with radioresistant tumours ($P = 0.0005$). There was also a significant difference in the 5-year local control rate of 85% and 63% for patients with radiosensitive and radioresistant tumours respectively ($P = 0.012$).

### Relationship between tumour vascularity and intrinsic radiosensitivity

Figure 1 shows the lack of correlation between measurements of IMD and SF2 in the same patient ($r = 0.17$, $P = 0.15$). In order to further check for the independence of the two parameters, bivariate log-rank analyses were carried out. Patients were stratified according to the median value for IMD after allowing for SF2 (Figures 2 and 3). The prognostic power of the IMD measurements increased and the *overall* significance levels for survival and local control were 0.006 and 0.013 respectively. Multivariate Cox regression analyses were also carried out which included: disease stage, patient age, SF2 and IMD (Table 2). SF2 emerged as the most important prognostic variable and after allowing for it only IMD was significantly associated with treatment outcome. Additional Cox analyses were made on a small subset of 42 patients for whom tumour grade and volume information were also available. For overall survival, IMD emerged as the most important prognostic parameter (relative risk (RR) of 5.2, $P = 0.022$) and after allowing for it only patient age was significant (RR of 3.1, $P = 0.031$). SF2 showed borderline significance ($P = 0.092$). For local control, SF2 was the most important parameter (RR of 3.8, $P = 0.016$) and after allowing for it, only IMD was significant (RR of 5.2, $P = 0.031$).

Although stage is often a powerful prognostic indicator for outcome, in this group of patients it was only of borderline significance for survival ($P = 0.085$) in univariate analysis but not significant on multivariate analysis. Stage was not a significant prognostic factor for local control in univariate or multivariate analysis.

### Combining IMD and SF2 measurements

As measurements of IMD and SF2 are completely independent, it is possible to combine the two factors and identify patients with good versus poor prognosis. In patients with radiosensitive tumours, there was no difference in survival for patients with low

© 1999 Cancer Research Campaign and highly vascular tumours (Figure 2). However, for patients with radioresistant tumours vascularity was very important (Figure 2). The 5-year survival levels for patients with radiosensitive and poorly vascularized versus radioresistant and well vascularized tumours were 77% and 18% respectively. Similarly, the 5-year local control levels for patients with radiosensitive and poorly vascularized versus radioresistant and well vascularized tumours were 90% and 50% respectively.

### DISCUSSION

Tumour vascularity measured as IMD has been shown by us to be a significant and independent prognostic factor for survival and local control following radiotherapy in 111 cervix cancer patients (Cooper et al, 1998). A smaller subset of 74 patients was studied here representing the 67% of patients for whom SF2 data were obtained. Reasons for not obtaining radiosensitivity results were either insufficient biopsy material or poor tumour growth in the assay. The smaller subset was representative of the larger study except that IMD was only of borderline significance for local control. This latter finding is probably due to the smaller number of patients included in the present study. The value of tumour SF2 as an independent prognostic factor for outcome following radiotherapy has been previously documented by us in 128 women with carcinoma of the cervix (West et al, 1993, 1997). The smaller cohort of 74 patients studies here was also representative of the larger group as SF2 was significantly associated with both survival and local control. The independence of the two biological parameters was shown in regression, bivariate log-rank and Cox multivariate analyses.

Although divergent mechanisms underlie inter-individual differences in tumour vascularity and radiosensitivity it is also possible that some common factors influence both parameters. Tumour angiogenesis is thought to be regulated by a balance between angiogenic promoters and inhibitors (Hanahan et al, 1996). Inter-tumour heterogeneity is a reflection of the positive and negative local effects of tumour, stromal and inflammatory cells acting in a paracrine fashion (Folkman, 1996). Tumour angiogenesis is stimulated by a number of growth factors including vascular endothelial growth factor (VEGF), basic and acidic growth factor and platelet-derived growth factor amongst others (Weidner et al, 1996), which in turn may be stimulated by local tumour characteristics such as hypoxia (Schweiki et al, 1992; Kuwabara et al, 1995). Furthermore, endothelial cells can stimulate the growth of tumour cells by the production of growth factors such as platelet-derived growth factor, insulin-like growth factor and interleukin-6 (Folkman, 1996). Hypoxia is also an important factor influencing cellular radiosensitivity (Thomlinson, 1968) and determining radiotherapy response (Hockel et al, 1996). The tumour suppressor gene, *TP53*, is another important modifier of intrinsic radiosensitivity (Bristow et al, 1996). It is of interest to note, therefore, that loss of wild-type *p53* has been shown to down-regulate the angiogenesis inhibitor, thrombospondin-1 (Dameron et al, 1994) and up-regulate VEGF (Rak et al, 1997).

Although IMD and SF2 are independent prognostic parameters, individual heterogeneity in tumour radiosensitivity dominates the differences in radiocurability seen for the cervix cancer patients studied here. This finding supports the results of a mathematical modelling study comparing clonogen number, radiosensitivity, hypoxia and proliferation measurements as predictors of radiotherapy response (Tucker et al, 1989). In Tucker's paper, SF2 was shown to dominate differences in radiocurability such that 'it may be necessary to correct for individual differences in intrinsic radiosensitivity before predictive assays based on other tumour characteristics might have any detectable clinical significance'. The main reason behind this is probably because the effect of SF2 is magnified over a course of protracted radiotherapy. For example, for a treatment involving 30 $\times$ 2 Gy fractions, tumours with mean SF2 values of 0.38 and 0.54 will have final differences in surviving fractions of around $10^{-8}$ and $10^{-12}$ respectively (Rak et al, 1997). In the work reported here we have shown that allowing for tumour SF2, increases the prognostic significance of IMD measurements. In addition, we have already shown a similar effect for patient age, tumour size and p53 expression (West et al, 1995).

Finally, we have shown the potential of combining two independent prognostic factors to define groups with large differences in outcome probabilities. Clearly, IMD appears more significant for radioresistant tumours. Therefore trials of anti-angiogenic chemotherapy could be directed at patients with radioresistant tumours, which would increase any chance of detecting a clinically significant difference.

In conclusion, the work reported here has shown no relationship between measurements of vascularity and radiosensitivity in the same tumour, and that allowing for SF2 increases the prognostic significance of IMD. This study emphasizes the potential of combining biological prognostic information to provide highly significant differences in radiotherapy outcome probabilities that may be of future clinical use to individualize patient treatment.

### ACKNOWLEDGEMENTS

This work was supported by the Cancer Research Campaign UK. Discussions with Professor Jolyon Hendry are gratefully acknowledged.

### REFERENCES

Awwad HK, El Naggar M, Mocktar and Barsoum M (1986) Intercapillary distance measurements as an indicator of hypoxia in carcinoma of the cervix uteri. *Int J Radiat Oncol Biol Phys* **12**: 1329–1333

Bristow RG, Benchimol S and Hill RP (1996) The p53 gene as a modifier of intrinsic radiosensitivity: implications for radiotherapy. *Radiother Oncol* **40**: 197–223

Cooper RA, Wilks DP, Logue JP, Davidson SE, Hunter RD, Roberts SA and West CML (1998) Tumour angiogenesis correlates with survival in carcinoma of the cervix treated with radiotherapy. *Clin Cancer Res* **4**: 2795–2800

Dameron KM, Volpert OV, Tainsky MA, Bouck N (1994) Control of angiogenesis in fibroblasts by p53 regulation of thrombospondin-1. *Science* **265**: 1582–1584

Davidson SE, Ngan R, Wilks DP, Moore JV and West CML (1994) A comparison of four methods for assessing tumour vascularity in carcinoma of the cervix. *Int J Oncol* **5**: 639–645

Dinh TV, Hannigan EV and Smith ER (1996) Tumour angiogenesis as a predictor of recurrence in stage Ib squamous cell carcinoma of the cervix. *Obstet Gynecol* **87**: 751–754

Eifel PJ, Berek JT and Thigpen JT (1993) Cancer of the cervix, vagina and vulva. In: *Cancer: Principles and Practice of Oncology*, 4th edn. Hoskins WJ, Perez CA, Young RC, DeVita VT, Hellman S and Rosenberg SA (eds), pp. 1152–1225. Lippincott: Philadelphia

Folkman J (1990) What is the evidence that tumours are angiogenesis dependent? *J Natl Cancer Inst* **82**: 4–6

Folkman J (1996) Tumour angiogenesis and tissue factor. *Nat Med* **2**: 167–168

Hanahan D and Folkman J (1996) Patterns and emerging mechanisms of the angiogenic switch during tumorigenesis. *Cell* **86**: 353–364

Hockel M, Schlenger K, Aral B, Mitze M, Schaffer U and Vaupel P (1996) Association between tumour hypoxia and malignant progression in advanced carcinoma of the uterine cervix. *Cancer Res* **56**: 4509–4515

© 1999 Cancer Research Campaign 

Hunter RD (1991) Female genital tract. In: *The Radiotherapy of Malignant Disease*, 2nd edn. Pointon CS (ed), pp. 279–308. Springer-Verlag: Berlin

Kapp DS and Giaccia AJ (1996) New directions for radiation biology research in the uterine cervix. *Mongr Natl Cancer Inst* **21**: 131–139

Kolstad P (1968) Intercapillary distance, oxygen tension and local recurrence in cervix cancer. *Scand J Clin Lab Invest* **106**: 145–157

Kuwabara K, Ogawa S, Matsumoto M, Koga S, Clauss M, Pinsky D, Lyn P, Leavy J, Witte L, Joseph-Silverstein J, Furie M, Torcia G, Cozzolino F, Kamada T and Stern DM (1995) Hypoxia-mediated induction of acidic/basic fibroblast growth factor and platelet-derived growth factor in mononuclear phagocytes stimulates growth of hypoxic endothelial cells. *Proc Natl Acad Sci USA* **92**: 4606–4610

Lindegaard JC, Overgaard J, Bentzen SM and Pedersen D (1996) Is there a radiological basis for improving the treatment of advanced stage cervical cancer? *Mongr Natl Cancer Inst* **21**: 105–112

Rak J and Kerbel RS (1997) bFGF and tumour angiogenesis: back in the limelight? *Nat Med* **3**: 1083–1084

Schweiki D, Itin A, Soffer D and Keshet E (1992) Vascular endothelial growth factor induced by hypoxia may mediate hypoxia-initiated angiogenesis. *Nature* **359**: 843–845

Siracka E, Siracky J, Pappova N and Revesz L (1982) Vascularisation and radiocurability in cancer of the uterine cervix. A retrospective study. *Neoplasma* **29**: 183–188

Thomlinson RH (1968) Changes of oxygenation in tumours in relation to irradiation. *Front Radiat Ther Oncol* **3**: 109–121

Tucker SL and Thames HD (1989) The effect of patient-to-patient variability on the accuracy of predictive assays of tumour response to radiotherapy: a theoretical evaluation. *Int J Radiat Oncol Biol Phys* **17**: 145–157

Vermeulen PB, Gasparini G, Fox SB, Toi M, Martin L, McCulloch P, Pezzella F, Viale G, Weidner N, Harris AL and Dirix LY (1996) Quantification of angiogenesis in solid human tumours: an international consensus on the methodology and criteria of evaluation. *Eur J Cancer* **32A**: 2474–2484

Weidner N and Folkman J (1996) Tumoural vascularity as a prognostic factor in cancer. In: *Important Advances in Oncology*, DeVita VT, Hellman S and Rosenberg SE (eds), pp. 167–190. Lippincott-Raven: Philidelphia.

Weidner N, Semple JP, Welch WR and Folkman J (1991) Tumour angiogenesis and metastasis-correlation in invasive breast carcinoma. *N Engl J Med* **324**: 1–8

West CML, Davidson SE, Roberts SA and Hunter RD (1993) Intrinsic radiosensitivity and prediction of patient response to radiotherapy for carcinoma of the cervix. *Br J Cancer* **68**: 819–823

West CML, Davidson SE, Roberts SA and Bhunter RD (1997) The independence of intrinsic radiosensitivity as a prognostic factor for patient response to radiotherapy of carcinoma of the cervix. *Br J Cancer* **76**: 1184–1190

West CML, Cooper SE, Davidson SE, Logue JP and Hunter RD (1998) Optimising measurements of tumour radiosensitivity. In: *Progress in Radio-oncology*, VI, Kogelink HD and Sedlmayer E (eds), pp. 517–524. Monduzzi Editore: Bologna.

Wiggens DL, Granai CO, Steinhoff MM and Calabresi P (1995) Tumour angiogenesis as a prognostic factor in cervical carcinoma. *Gynecol Oncol* **56**: 353–356

*© 1999 Cancer Research Campaign*

## References

[PDF_00409] Awwad H. K., el Naggar M., Mocktar N., Barsoum M. (1986). Intercapillary distance measurement as an indicator of hypoxia in carcinoma of the cervix uteri.. Int J Radiat Oncol Biol Phys.

[PDF_00412] Bristow R. G., Benchimol S., Hill R. P. (1996). The p53 gene as a modifier of intrinsic radiosensitivity: implications for radiotherapy.. Radiother Oncol.

[PDF_00415] Cooper R. A., Wilks D. P., Logue J. P., Davidson S. E., Hunter R. D., Roberts S. A., West C. M. (1998). High tumor angiogenesis is associated with poorer survival in carcinoma of the cervix treated with radiotherapy.. Clin Cancer Res.

[PDF_00418] Dameron K. M., Volpert O. V., Tainsky M. A., Bouck N. (1994). Control of angiogenesis in fibroblasts by p53 regulation of thrombospondin-1.. Science.

[PDF_00423] Dinh T. V., Hannigan E. V., Smith E. R., Hove M. J., Chopra V., To T. (1996). Tumor angiogenesis as a predictor of recurrence in stage Ib squamous cell carcinoma of the cervix.. Obstet Gynecol.

[PDF_00432] Folkman J. (1996). Tumor angiogenesis and tissue factor.. Nat Med.

[PDF_00430] Folkman J. (1990). What is the evidence that tumors are angiogenesis dependent?. J Natl Cancer Inst.

[PDF_00433] Hanahan D., Folkman J. (1996). Patterns and emerging mechanisms of the angiogenic switch during tumorigenesis.. Cell.

[PDF_00435] Hockel M., Schlenger K., Aral B., Mitze M., Schaffer U., Vaupel P. (1996). Association between tumor hypoxia and malignant progression in advanced cancer of the uterine cervix.. Cancer Res.

[PDF_00442] Kapp D. S., Giaccia A. J. (1996). New directions for radiation biology research in cancer of the uterine cervix.. J Natl Cancer Inst Monogr.

[PDF_00444] Kolstad P. (1968). Intercapillary distance, oxygen tension and local recurrence in cervix cancer.. Scand J Clin Lab Invest Suppl.

[PDF_00446] Kuwabara K., Ogawa S., Matsumoto M., Koga S., Clauss M., Pinsky D. J., Lyn P., Leavy J., Witte L., Joseph-Silverstein J. (1995). Hypoxia-mediated induction of acidic/basic fibroblast growth factor and platelet-derived growth factor in mononuclear phagocytes stimulates growth of hypoxic endothelial cells.. Proc Natl Acad Sci U S A.

[PDF_00451] Lindegaard J. C., Overgaard J., Bentzen S. M., Pedersen D. (1996). Is there a radiobiologic basis for improving the treatment of advanced stage cervical cancer?. J Natl Cancer Inst Monogr.

[PDF_00454] Rak J., Kerbel R. S. (1997). bFGF and tumor angiogenesis--back in the limelight?. Nat Med.

[PDF_00456] Shweiki D., Itin A., Soffer D., Keshet E. (1992). Vascular endothelial growth factor induced by hypoxia may mediate hypoxia-initiated angiogenesis.. Nature.

[PDF_00459] Siracká E., Siracký J., Pappová N., Révész L. (1982). Vascularization and radiocurability in cancer of the uterine cervix. A retrospective study.. Neoplasma.

[PDF_00464] Tucker S. L., Thames H. D. (1989). The effect of patient-to-patient variability on the accuracy of predictive assays of tumor response to radiotherapy: a theoretical evaluation.. Int J Radiat Oncol Biol Phys.

[PDF_00467] Vermeulen P. B., Gasparini G., Fox S. B., Toi M., Martin L., McCulloch P., Pezzella F., Viale G., Weidner N., Harris A. L. (1996). Quantification of angiogenesis in solid human tumours: an international consensus on the methodology and criteria of evaluation.. Eur J Cancer.

[PDF_00471] Weidner N., Folkman J. (1996). Tumoral vascularity as a prognostic factor in cancer.. Important Adv Oncol.

[PDF_00474] Weidner N., Semple J. P., Welch W. R., Folkman J. (1991). Tumor angiogenesis and metastasis--correlation in invasive breast carcinoma.. N Engl J Med.

[PDF_00476] West C. M., Davidson S. E., Roberts S. A., Hunter R. D. (1993). Intrinsic radiosensitivity and prediction of patient response to radiotherapy for carcinoma of the cervix.. Br J Cancer.

[PDF_00479] West C. M., Davidson S. E., Roberts S. A., Hunter R. D. (1997). The independence of intrinsic radiosensitivity as a prognostic factor for patient response to radiotherapy of carcinoma of the cervix.. Br J Cancer.

[PDF_00486] Wiggins D. L., Granai C. O., Steinhoff M. M., Calabresi P. (1995). Tumor angiogenesis as a prognostic factor in cervical carcinoma.. Gynecol Oncol.

